# A bacterial outer membrane vesicle-based click vaccine elicits potent immune response against *Staphylococcus aureus* in mice

**DOI:** 10.3389/fimmu.2023.1088501

**Published:** 2023-01-19

**Authors:** Jingjing Sun, Xuansheng Lin, Yige He, Baozhong Zhang, Nan Zhou, Jian-dong Huang

**Affiliations:** ^1^ CAS Key Laboratory of Quantitative Engineering Biology, Shenzhen Institute of Synthetic Biology, Shenzhen Institutes of Advanced Technology, Chinese Academy of Sciences, Shenzhen, Guangdong, China; ^2^ ZJU-Hangzhou Global Scientific and Technological Innovation Center, Zhejiang University, Hangzhou, China; ^3^ School of Biomedical Sciences, Li Ka Shing Faculty of Medicine, University of Hong Kong, Hong Kong, Hong Kong SAR, China; ^4^ Department of Clinical Oncology, Shenzhen Key Laboratory for Cancer Metastasis and Personalized Therapy, The University of Hong Kong-Shenzhen Hospital, Shenzhen, Guangdong, China; ^5^ Guangdong-Hong Kong Joint Laboratory for RNA Medicine, Sun Yat-Sen University, Guangzhou, China

**Keywords:** outer membrane vesicles, *Staphylococcus aureus* vaccine, SpyCatcher-SpyTag, ‘click’ display, flexible antigen display, multi-targeting vaccine

## Abstract

*Staphylococcus aureus* infection is a severe public health concern with the growing number of multidrug-resistant strains. *S. aureus* can circumvent the defense mechanisms of host immunity with the aid of multiple virulence factors. An efficacious multicomponent vaccine targeting diverse immune evasion strategies developed by *S. aureus* is thus crucial for its infection control. In this study, we exploited the SpyCatcher-SpyTag system to engineer bacterial outer membrane vesicles (OMVs) for the development of a multitargeting *S. aureus* click vaccine. We decorated OMVs with surface exposed SpyCatcher *via* a truncated OmpA(a.a 1-155)-SpyCatcher fusion. The engineered OMVs can flexibly bind with various SpyTag-fused *S. aureus* antigens to generate an OMV-based click vaccine. Compared with antigens mixed with alum adjuvant, the click vaccine simultaneously induced more potent antigen-specific humoral and Th1-based cellular immune response, which afforded protection against *S. aureus* Newman lethal challenge in a mouse model. Our study provided a flexible and versatile click vaccine strategy with the potential for fighting against emerging *S. aureus* clinical isolates.

## 1 Introduction

*Staphylococcus aureus* (*S. aureus*) is a Gram-positive bacterium commonly found as part of the normal flora on the skin of humans (1). It usually becomes invasive in patients with immunological or barrier defects, and has been a leading cause of many infection diseases in the hospital and community (2–4). *S. aureus* infections are notoriously difficult to control as an increasing number of its clinical isolates exhibits resistance to multiple antibiotics (5–7). Patients recovered from *S. aureus* infection also show no immunity to subsequent infections (1). To this end, there is a dire need to develop prophylactic vaccines that provide protective immunity against *S. aureus*.

A variety of surface adhesion proteins and secreted proteins which are implicated in *S. aureus* immune evasion have been exploited as potential vaccine antigens (1, 8–10a; 11). The current antigenic targets mainly focus on virulence factors (10, 12, 13), capsular polysaccharide (14), iron-regulated proteins (15–17) and cell wall-anchored enzymes such as adenosine synthase (18). However, most of the clinical trials for vaccines based on antigens from either a single protein or a certain protein family have failed so far (19, 20). *S. aureus* can easily circumvent defense mechanisms of the host immune system by expressing multiple virulence factors and combined function of invasion molecules (21). Therefore, it still demands novel vaccine strategies which combine multiple antigenic components to simultaneously target diverse strategies that *S. aureus* deployed to circumvent host immunity.

Outer membrane vesicles (OMVs) are membrane-derived vesicles released spontaneously during growth by many bacteria species (22, 23). As natural OMVs obtained from a pathogen present a range of surface antigens in a native conformation, they have been directly explored as vaccine products against parental pathogenic bacteria (24, 25). More recently, due to the immunostimulatory, self-adjuvant and ease of genetic manipulation properties, engineered OMVs show promise to become a versatile vaccine platform (26–28). OMV-based vaccines were initially generated by directly fusing OMV scaffold protein with antigens or routing modified antigens to the OMV lumen (29–34). More recently, by combining the SpyCatcher-SpyTag system, the function of OMVs as nanocarriers was further expanded for rapid and flexible surface display of recombinant proteins or tumor antigens (35–37). The covalent bond formation between SpyCatcher and SpyTag can occur at a range of temperatures and pH values (38). Such a flexible “click” system should allow rapid and multiple antigen attachment to OMVs *in vitro*, which will enable an effective and multi-targeting *S. aureus* vaccine platform.

In this study, we engineered OMVs secreted from a common laboratory *Escherichia coli* (*E. coli*) MG1655 strain with surface exposed SpyCatcher *via* truncated OmpA-SpyCatcher fusions. We show that the engineered OMVs can flexibly assemble with various SpyTag-fused *S. aureus* antigens to generate OMV-based click vaccines. The click vaccine simultaneously induced strong humoral and cellular immunity specific for multiple antigens displayed, which conferred protection against *S. aureus* lethal challenge in a mouse model. Our results presented a multipurpose and potent OMV-based *S. aureus* vaccine platform.

## 2 Materials and methods

### 2.1 Bacterial strains, plasmids and media

Bacteria strains and plasmids used in this study are listed in **Supplementary Table 1**. *E. coli* MG1655 wild type (WT) strain was utilized as a parental strain for mentioned isogenic gene deletion and chromosomal modification. *E. coli* strain BL21(DE3) was used for recombinant protein expression. Standard Luria-Bertani (LB) broth with appropriate antibiotics (100 μg/ml ampicillin, 50 μg/ml kanamycin or 25 μg/ml chloramphenicol) was used for *E. coli* culture.

### 2.2 Strain construction

To create the *lpxM* deletion strain, PCR-amplified DNA fragments containing 40 bp homologous arms flanking *lpxM* open reading frame together with a selection antibiotic marker were purified. PCR products were then electroporated into MG1655 harboring λ red recombinase expression plasmid pKD46. Positive clones were selected by 25 μg/ml chloramphenicol and verified by DNA sequencing. Subsequently, the antibiotic selection marker flanked by loxP sites was removed using the helper plasmid p705Cre. The Δ*lpxM ompA-spycatcher* strain was further constructed using the similar recombineering method. An *ompA*(1-465 bp)*-G4S linker-spycatcher-6his* fusion gene was synthesized and used to replace the chromosomal *ompA* gene. Positive clones were selected using 50 μg/ml kanamycin and confirmed by DNA sequencing.

### 2.3 Plasmid construction

A gene fragment encoding SpyTag (AHIVMVDAYKPTK) was first inserted in the pET28a plasmid between NheI and BamHI restriction sites to produce the pET28a-spytag plasmid. Gene fragments of GFP and *S. aureus* antigen rEsxA, rSbi, and a mutated form of rSpA_KKAA_ (rSpA) from the Newman strain were synthesized and inserted between BamHI and SalI restriction sites to create a series of SpyTag-fused GFP or antigen expression plasmids. Positive clones were screened and verified by colony PCR and DNA sequencing. Primers used in this study are listed in **Supplementary Table 2**. Correctly constructed plasmids were then prepared and transformed into BL21 (DE3) strain for protein expression and purification.

### 2.4 Purification of His_6_-tagged proteins

SpyTag fusion proteins were expressed in *E. coli* BL21 (DE3) strains and purified by nickel column. Bacteria were grown in LB broth at 37°C with constant shaking (220 r.p.m) until OD_600_ of 0.6 before adding 1 mM IPTG to induce fusion protein expression. After induction for an additional 3-6 h at 37°C, cells were harvested by centrifugation. Bacterial pellets were then resuspended in 2 ml of lysis buffer (0.5 M NaCl, 50 mM pH 7.2 Tris-HCl, 40% glycerol, 20 mM imidazole, 150 mM PMSF, and 500 mM DTT) followed by sonication for cell lysis. Bacterial lysates were obtained by centrifugation at 15,000 g for 30 min and the resultant supernatant was subjected to a 1.5-ml Ni-nitrilotriacetic acid-agarose column (Qiagen) equilibrated with binding buffer (0.5 M NaCl, 50 mM pH 7.2 Tris-HCl, 40% glycerol, and 40 mM imidazole). After rounds of washing with binding buffer, proteins were eluted with elution buffer (0.5 M NaCl, 50 mM pH 7.2 Tris-HCl, 40% glycerol, and 500 mM imidazole).

### 2.5 OMV purification and quantification

Engineered bacteria were grown in 250 ml LB broth at 37°C with constant shaking (220 r.p.m) until OD_600_ of 1.0. The bacteria culture was centrifuged at 4,500 × *g* for 20 min at 4°C. The resultant supernatant was filtered by 0.45-µm filters (Corning) and followed by ultracentrifugation at 100,000 × *g* for 1 h at 4°C. OMV-containing pellets were then resuspended in DPBS and filtered by 0.22-µm filters (Corning) for further use. The total protein concentration of purified OMVs was determined by BCA assay. The surface display of SpyCatcher on OMVs was verified by western blot using a His-Tag Monoclonal antibody (Proteintech). The OMV size was determined by Nano-flow cytometry (NanoFCM) according to manufacture instructions.

### 2.6 Transmission electron microscopy

For TEM analysis, diluted OMVs were fixed with 4% PFA and absorbed to copper grids. Negative staining was performed with 1% aqueous uranyl acetate at room temperature and washed to remove excess aqueous uranyl acetate. Electron micrographs were collected from randomly-selected fields using Philips CM100 transmission electron microscope.

### 2.7 SpyCatcher and SpyTag reaction assay

Equal amount (50 μg) of OMV-SpyCatcher and SpyTag-GFP proteins were incubated at room temperature for 30 min. Unbound SpyTag-GFP in the reaction were removed by ultrafiltration using a 0.5mL 100-KDa ultrafiltration unit (Millipore). For the first round of ultrafiltration, the reaction was supplemented to a volume of 300 μl in total by adding DPBS and then centrifuged at 14,000 g until a volume of 50 μl. The resultant reaction was resuspended to 300 μl for the second round of ultrafiltration to a volume of 50 μl. GFP fluorescence was captured by UVP ChemStudio (Analytikjena). To perform the reaction assay in living cells, Δ*lpxM* and Δ*lpxM ompA-spycatcher* bacterial cells were collected and washed in PBS. Bacteria were then adjusted to OD_600_ of 0.3 and incubated with or without 5 μg Spytag-GFP at room temperature for 10 min. Samples were washed three times with ice-cold PBS and subjected to flow cytometry analysis using FITC channel (CytoFLEX, Beckman).

### 2.8 Animal immunization

Six-week-old female BALB/c mice were immunized with OMV triple-linked antigens, a cocktail of three OMV single-linked antigens and antigens formulated with 10% aluminum hydroxide gel (AHG) by subcutaneous (s.c) injection. For OMV triple-linked antigens group, 50 μg OMV-SpyCatcher were incubated with three antigens (10 μg each) for 30 min. For a cocktail of three OMV single-linked antigens group, each 16.7 μg OMV-SpyCatcher were incubated with 10 μg of a single antigen for 30 min, then the three OMV single-linked antigens were pooled together to make a cocktail before injection. To prepare AHG+Antigens group, 30 μg antigens (10 μg for each) were mixed with 10% AHG. 50 μg empty OMVs purified from Δ*lpxM* strain and PBS were also injected as control groups. Mice were immunized three times at 2-week interval. Seven days after the last immunization, blood samples or spleens were collected for ELISA or ELISpot assay respectively. Animal immunization experiments were performed in accordance with institutional guidelines following experimental protocol review and approval by the Institutional Animal Care and Use Committee.

### 2.9 Antibody detection by ELISA

The IgG detection were performed by enzyme-linked immunosorbent assay (ELISA). SpyTag fused EsxA, tSbi and SpA proteins (1 µg/ml in 0.05 M carbonate-bicarbonate buffer, pH 9.6, 200 µl/well) were coated in ELISA plates (Nunc, Denmark) and incubated overnight at 4°C. The plates were blocked with phosphate-buffered saline (PBS) containing 5% nonfat milk at room temperature for 3 h and washed four times with PBS containing 0.05% Tween 20. Three-fold serially diluted mice sera were added into the wells and incubated at room temperature for 1 h. Then the plates were washed six times with PBS containing 0.05% Tween 20 and incubated with horseradish peroxidase (HRP)-conjugated goat anti-mouse IgG at room temperature for 1 h. The color was developed using tetramethylbenzidine (TMB) solution (Sigma) and absorbance was measured using a plate reader (BioTek Microplate Reader) at 450 nm. The antibody level was determined as the area under the curve (AUC) calculated by GraphPad Prism 9 (GraphPad Software, USA).

### 2.10 ELISpot assay

IFN-γ-producing splenocytes from vaccinated or naive unvaccinated mice were analyzed using a Mouse IFN-γ ELISpot ^PLUS^ (HRP) kit (Mabtech). Spleens were minced in RPMI 1640 Medium (Thermo Fisher Scientific) and cells were filtered through a 70-μm sterile cell strainer (Corning). Red blood cells were lysed using red blood cell lysis buffer. Remaining splenocytes were washed and adjusted to a concentration of 1 × 10^6^ cells ml^-1^ in complete RPMI 1640 medium. 100 μl cells/well were added to conditioned ELISpot plates and each recombinant antigen, stSpA, stSbi or stEsxA, was used as the inducer with a final concentration of 2 μg/ml. Plates were incubated at 37°C in a humidified incubator with 5% CO_2_ for 24 hours. Following 2 h primary antibody incubation and 1 h secondary antibody incubation at room temperature, spots were developed using TMB substrate solution and recorded in an immunospot analyzer.

### 2.11 Lethal challenge

Immunized animals were challenged on day 42 by intravenous injection with 5 × 10^7^ CFU of *S. aureus* Newman strain. The well-being of infected mice was monitored daily for 6 days and lethal disease was recorded. The surviving mice were euthanized after day 6 post challenge as a predetermined humane endpoint. Lethal challenge experiments were approved by the Committee on the Use of Live Animals in Teaching and Research of the University of Hong Kong (approval no. CULATR 5163-19).

### 2.12 Statistical analysis

One way ANOVA was used to analyze the statistical significance of ELISpot assay results and GFP binding. Log rank (Mantel-Cox) analysis was used to analyze the statistical significance of the data from the lethal-challenge experiments. Analysis was performed using GraphPad Prism 9 (GraphPad Software, USA), and a P value of <0.05 was considered statistically significant.

## 3 Results

### 3.1 Generation and characterization of engineered bacterial OMVs

**Figure 1:**
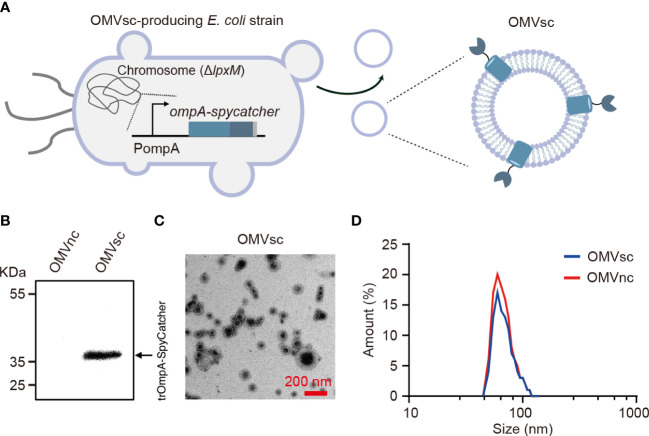
Generation and characterization of engineered bacterial OMVs. **(A)** A schematic diagram of the Δ*lpxM ompA-spycatcher E coli* strain producing engineered OMVs containing trOmpA (a.a 1-155)-SpyCatcher fusions. **(B)** Western blot analysis of OMVs purified from Δ*lpxM ompA-spycatcher* (OMVsc) and the Δ*lpxM* strain (OMVnc). An anti-His_6_ primary antibody was used to detect the trOmpA-SpyCatcher-His_6_ fusion protein. **(C)** A randomly selected transmission electron microscopy (TEM) image of OMVsc. Scale bar: 200 nm. **(D)** Nano-flow cytometry analysis of the size distribution of OMVsc and OMVnc.

We modified the composition of bacterial OMVs by deleting the *lpxM* gene which encodes lipid A acyltransferase in *E. coli* MG1655. The deletion of *lpxM* leads to under-acylation of LPS and largely attenuates the endotoxic activity of LPS which may cause adverse effects for *in vivo* applications of engineered OMVs (39–41). To further generate OMVs with surface-exposed SpyCatcher, we did genomic insertion in the Δ*lpxM* strain to replace the chromosomal *ompA* open reading frame with a truncated *ompA (*1- 465 bp*)* and *spycatcher* fusion gene (**Figure 1A**). OmpA is one of the most abundant outer membrane proteins in *E. coli* and subsequently in OMVs (42). It was reported that the 144-160 amino acids of OmpA were extracellular exposed (43) and fusion with a truncated version of OmpA (a.a 1-159) allowed the display of a fused protein on *E. coli* cell surfaces (44, 45). Therefore, the SpyCatcher is expected to be exposed on the surface of engineered OMVs when fused with the 155 a.a truncated OmpA (trOmpA) (**Figure 1A**).

We first detected the expression of the trOmpA-SpyCatcher fusion protein by western blot in engineered OMVs (OMVsc) isolated from the Δ*lpxM ompA-spycatcher* strain, but not in control OMVs (OMVnc) from the Δ*lpxM* strain (**Figure 1B**). We confirmed the OMV morphology by TEM as electron-dense particles with characteristic OMV structures (**Figure 1C**). We further determined the size of purified OMVs by Nano-flow cytometry. **Figure 1D** showed the size of OMVsc displayed a similar pattern as that of OMVnc with a typical diameter distribution ranging from 40 to 200 nm, which is in accordance with previous findings (46–48).

### 3.2 ‘Click’ display of GFP on Engineered OMV

**Figure 2:**
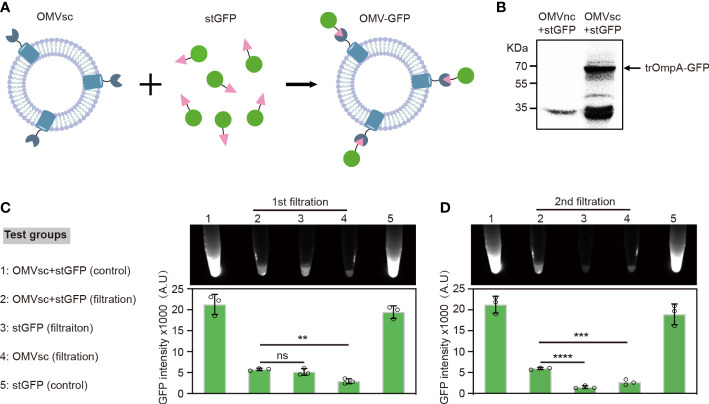
Click display of GFP on engineered OMVs. **(A)** A schematic diagram of the click display of GFP on the surface of OMVsc. **(B)** Detection of the assembly between OMVsc and stGFP by western blot. OMVsc and OMVnc were incubated with stGFP separately and unbond stGFP was washed subsequently. The trOmpA-GFP fusion protein was detected by using an anti-His_6_ primary antibody. **(C)** Detection of the fluorescence of OMV-GFP. OMVsc were incubated with stGFP at room temperature to allow *in vitro* binding. The mixing sample, sfGFP and OMVsc alone were subjected to two rounds of ultrafiltration. Fluorescent images were taken after each around of ultrafiltration and fluorescent intensity was quantitatively analyzed (n=3). An OMVsc and stGFP mixing group and a stGFP group were used as non-ultrafiltration control. The data are shown as mean ± SD. Statistical analysis was performed by one way ANOVA. ns: not significant; **P <0.01; ***P< 0.001; ****P < 0.0001.

To examine whether the OMV-presented SpyCatcher based on trOmpA fusion works well *in vitro*, we applied SpyTag-fused GFP (stGFP) to test the ability of ‘click’ display of heterogenous antigens by our OMVsc (**Figure 2A**). OMVsc and OMVnc were separately incubated with stGFP and the ‘click’ linking of OMV and GFP was analyzed by western blot. The result indicated that only OMVsc group but not OMVnc generated trOmpA-GFP fusion proteins (~70 kDa), proving specific conjugation between OMVsc and stGFP mediated by SpyCatcher-SpyTag system (**Figure 2B**).

We further tested the specific binding of stGFP on OMVsc by fluorescence. When incubating living bacterial cells with stGFP, fluorescence signal could only be detected by flow cytometry for the strain expressing trOmpA-SpyCatcher after washing (**Supplementary Figure 1**). We then incubated purified OMVsc with stGFP and subsequently removed the unbound stGFP by ultrafiltration. After a second round of ultrafiltration, we detected significantly higher GFP fluorescence in OMVsc+stGFP filtration group compared with either stGFP or OMVsc group, suggesting the trap of stGFP by OMVst during ultrafiltration (**Figure 2C**). Together, our results demonstrate that engineered OMVsc could utilize trOmpA-SpyCatcher to ‘click’ display SpyTag fused GFP, which makes a versatile antigen display vaccine platform.

### 3.3 Decoration of engineered OMVs with heterologous *S. aureus* antigens

**Figure 3:**
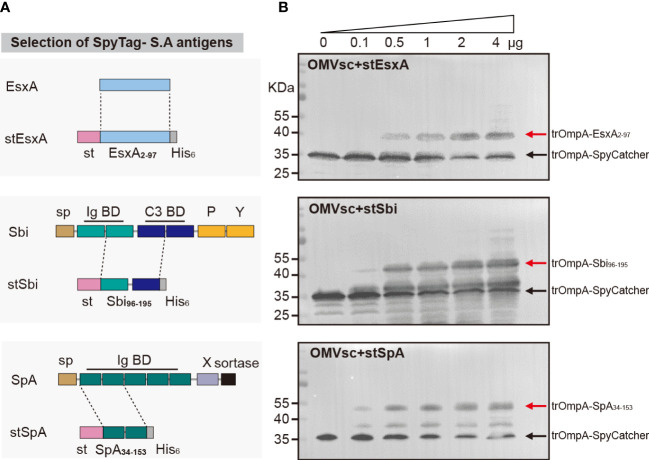
Decoration of engineered OMVs with heterologous *S. aureus* antigens. **(A)** Schematic representation of the selection of the recombinant *S. aureus* antigen stEsxA, stSbi and stSpA. **(B)** Western blot analysis of OMVsc bond with heterologous recombinant *S. aureus* antigens by using an anti-His_6_ primary antibody. The red arrow indicates the trOmpA-antigen conjugations and the black arrow indicates the trOmpA-SpyCather fusion protein.

We selected three *S. aureus* antigens which are conserved in a large panel of *S. aureus* strains to assemble with OMVsc. The three antigens, EsxA, truncated Sbi and a mutated form of truncated SpA (SpA_KKAA_) (49), target different virulent or immune evasion pathways of *S. aureus.* We fused SpyTag with the entire EsxA (a.a 2-97), one Ig binding domain and one complement binding domain of Sbi (a.a 96-195) and two Ig binding domains of SpA (a.a 34-153) to separately generate stEsxA, stSbi and stSpA antigens (**Figure 3A**). To test whether the recombinant antigens could be displayed on OMVsc, we incubated 10 μg of OMVsc with various amounts of stEsxA, stSbi, or stSpA antigens and then detected the OMV-antigen binding using western blot. As expected, all three SpyTag fused antigens formed conjugation with OMVsc in a dose-dependent manner (**Figure 3B**).

### 3.4 Immunogenicity of OMV-based *S. aureus* click vaccine in mice

**Figure 4:**
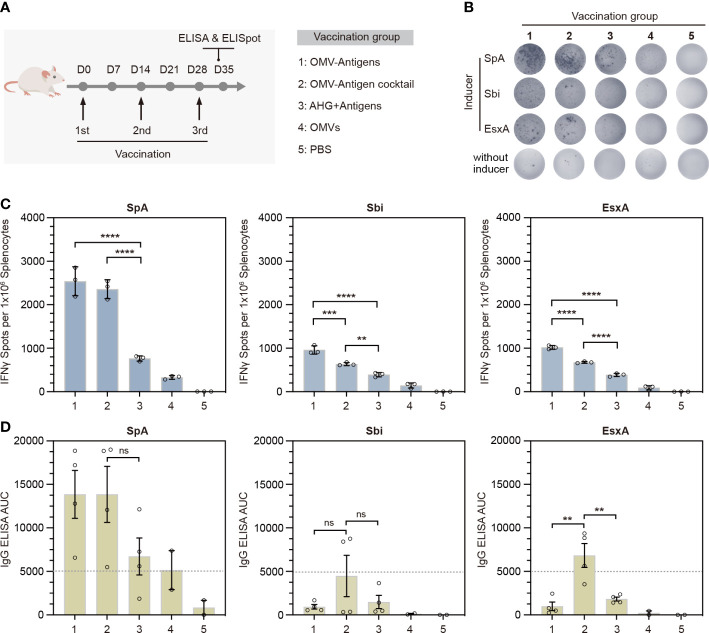
*In vivo* immunogenicity test of the OMV-based *S. aureus* click vaccine. **(A)** Schematic representation of the vaccination strategy and each vaccination group. **(B)** Representative images of splenic IFN-γ ELISpot responses for each immunization group. **(C)** Quantification of IFN-γ positive splenocytes for each immunization group. Splenocytes were prepared from spleens taken from mice (n=3) 7 days post the third immunization. **(D)** Quantification of antigen-specific IgG for each immunization group by ELISA in terms of the area under the curve (AUC). Sera were taken from mice (n=4) for OMV-Antigens, OMV-Antigen cocktail and AHG+Antigens immunization groups and mice (n=2) from OMV and PBS immunization group 7 days post the third immunization. The data are shown as mean ± SEM. Statistical analysis was performed by one way ANOVA. ns, not significant. **P <0.01; ***P <0.001; ****P <0.0001.

To evaluate the immunogenicity of our click vaccine *in vivo*, we immunized mice subcutaneously with 3 doses of different vaccines (**Figure 4A**). In particular, we prepared two forms of click vaccines, OMV-Antigens (OMVsc triple-linked antigens) and OMV-Antigen cocktail (a cocktail of three OMVsc single-linked antigen). The click vaccines were compared with AHG+antigens (Antigens mixed with commonly used adjuvant AHG), OMVs (OMVsc alone) and PBS as a negative control.

Th1-biased cellular immunity has been shown to play essential roles in the protection against *S. aureus*. The release of IFN-γ is indicative of Th1-biased immune responses. Therefore, we performed IFN-γ ELISpot assays for splenocytes collected from immunized mice 7 days after the third immunization. The results showed that splenocytes from both click vaccine groups had significantly more IFN-γ secreting cells than that of AHG+antigens and other control groups (**Figures 4B, C**). Quantification of IFN-γ^+^ spots indicated that OMV-Antigens induced stronger anti-Sbi and anti-EsxA cellular immune response than OMV-Antigen cocktail (**Figure 4C**).

To investigate the stimulation of humoral immunity by our click vaccine, we also collected the serum samples from each group to detect the levels of antigen-specific immunoglobulin G (IgG) by ELISA in terms of area under the curve (AUC). The results showed that immunization with OMV-Antigen cocktail induced high levels of IgG for all antigens, while immunization with OMV-Antigens only induced a high level of IgG for SpA. The anti-Sbi and anti-EsxA IgG induced by OMV-Antigens were comparable with that induced by AHG+antigens (**Figure 4D**). Taken together, the above results suggested that the OMV-based click vaccine could successfully evoke potent *S. aureus* antigen-specific humoral and cellular immunity in a mouse model.

### 3.5 Protective effects of OMV-based click vaccine against *S. aureus* lethal challenge in mice

**Figure 5:**
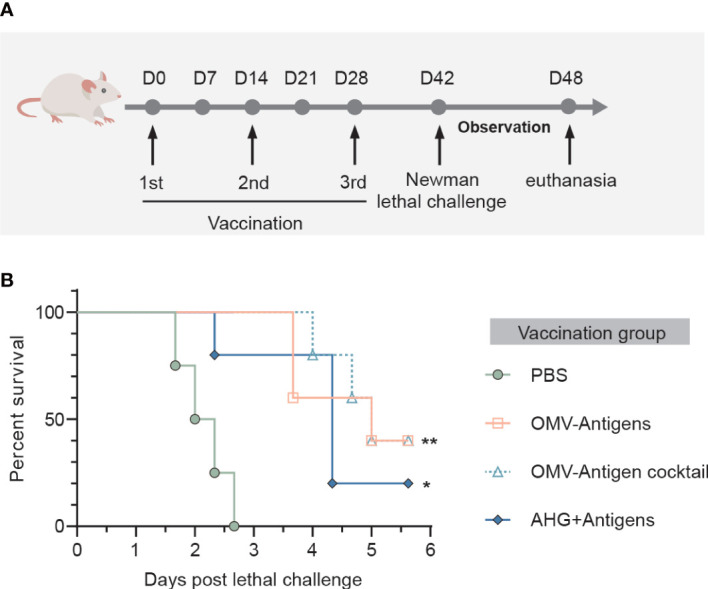
Immunization with the OMV-based click vaccine generated protective immunity against lethal challenge with *S. aureus* Newman. **(A)** Mice (n=5) were immunized with OMV-Antigens, OMV-Antigen cocktail, Antigens mixed with 10% AHG and PBS as a control. Lethal dose of *S. aureus* Newman was injected intravenously 7 days post the third immunization and the survival of mice were monitored afterwards. **(B)** The survival curve of the mice in different immunization groups. Statistical analysis was performed by the Log-rank (Mantel-Cox) test. *P < 0.05; **P < 0.01.

We then investigated whether immune responses elicited by the click vaccine could translate into protective effects against *S. aureus* lethal challenge in mice. Mice receiving different vaccinations were challenged with a lethal dose of *S. aureus* Newman strain (**Figure 5A**). All mice in the PBS treated group died within 72 hours post Newman challenge. However, survival rates were significantly improved by vaccination with OMV-Antigens (40% survival; *P=*0.0027) or OMV-Antigen cocktail (40% survival; *P=*0.0027). Notably, our click vaccines also conferred better protection compared with AHG+antigens vaccination (20% survival; *P=*0.0150) (**Figure 5B**).

## 4 Discussion

*S. aureus* infection is a growing public health concern due to the emergence of multidrug-resistant (MDR) strains. Vaccination holds promise to be an alternative strategy to control MDR-pathogen infections. In this work, we exploited the “Plug-and-Display” technology to decorate OMVs with heterologous antigens for *S. aureus* vaccine development. We generated an OMV-based multi-targeting click vaccine which elicited stronger humoral and cellular immune responses compared with that evoked by the alum adjuvanted vaccine. The click vaccine showed potent protective activity against *S. aureus* Newman lethal challenge with increased survival rate in mice.

*S. aureus* utilizes a myriad of virulence factors to circumvent the defense mechanisms of the host immune system (50). Subunit vaccines composed of antigens targeting an individual pathway may not be ideal, as the redundant and multifaceted functions of virulence factors can compensate for the loss of function of a particular vaccine target (1a). Therefore, it is crucial to combine multiple antigens targeting diverse immune evasion strategies exploited by *S. aureus* to develop an efficacious vaccine. In a previous study, a successful OMV-based *S. aureus* vaccine platform was developed by harnessing the lipoprotein transport machinery to enrich multiple lipidated antigens within OMVs (34). This strategy requires an individual co-expression and purification process for OMVs containing each antigen. Detoxified *S. aureus* membrane vesicles (MVs) which contain multiple native antigens have also been demonstrated as a promising vaccine candidate (51). Promoting the efficient release and detoxification of EVs demands sophisticated genetic engineering of a given *S. aureus* clinical isolate. Here, we showed that the OMV-based click display technology could facilitate the formulation of diverse selected antigens in a rapid and flexible fashion (**Figure 3B**). As the antigen combinations displayed on engineered OMVs can be easily adjusted by generating novel SpyTag-antigen fusions, our click vaccine strategy is valuable for protecting against emerging *S. aureus* clinical isolates when selected antigens were not conserved.

To engineer OMVs for surface display of proteins, a novel strategy is to fuse SpyCatcher or SpyTag with surface scaffold proteins of the OMV. For this purpose, hemoglobin protease (Hbp) and ClyA have already been exploited to load functional SpyTag or SpyCatcher on OMV surface (35–37). OmpA is also one of the most abundant surface scaffold proteins of bacteria OMVs. However, as the N- and C-terminal of OmpA are both on the periplasmic side of the outer membrane (52), fusion with OmpA would lead to the package of protein of interest into the lumen of OMVs (53). Notably, the 144-160 amino acids of OmpA were reported to be extracellularly exposed and recombinant proteins fused with a truncated version of OmpA (a.a 1-159) were displayed on *E. coli* cell surfaces (44, 45). This raises a potential site for the surface display of exogenous antigens on OMVs. In this study, we fused SpyCatcher to the C-terminal of a truncated OmpA (a.a 1-155). We found the formation and morphology of OMVs were not much affected by the fusion (**Figures 1C, D**). With *in-vitro* incubation, SpyTag fused GFP or *S. aureus* antigens could bind to the engineered OMVs (**Figures 2B, C**), suggesting that SpyCatcher was exposed on the OMV surface and functioned properly. Therefore, we provided another anchoring site for OMV surface display and expanded the engineerability of OMVs. When combined with additional orthogonal protein ligation systems such as split inteins (54), expanded OMV surface display sites may allow programmable loading of different antigens with precise spatiotemporal and ratio control. A potential limitation for this OMV-based click platform for vaccine development is the stability of the antigens to be linked. Antigens used for immunization are often from surface adhesion or membrane proteins. Their instability and tendency to aggregate will prevent their flexible linking with OMVs. For this type of antigen, a compromised way might be linking it with an alternative OMV scaffold protein *in vivo* by direct gene fusion or other orthogonal ligation systems such as split inteins. The resultant OMVs can then be isolated for further antigen loading.

Previous studies have shown that OMV-based vaccines usually elicited stronger immune responses compared with that evoked by purified antigens formulated with alum (30, 34). Our study also showed similar results that the OMV-based click vaccine, particularly the cocktail of OMV-antigen, could induce higher antigen-specific IgG production than that obtained with antigens formulated with AHG (**Figure 4D**). Our ELISpot results also confirmed that the OMV-based click vaccine could simultaneously induced significantly stronger T cell response for all antigens delivered. This may be explained by the observation that OMVs can be efficiently uptaken by antigen presenting cells (APCs) for subsequent T cell activation (37, 55). As AHG adjuvant tends to favor Th2 cytokine production and thus the antibody-based humoral response, it will be interesting to use Th1 adjuvant CpG for comparison to further evaluate the capacity of OMV-based vaccine to provoke Th1-biased T cell response. Noteworthy, “empty” OMVs also evoked detectable IFN-γ^+^ cellular response (**Figure 4C**, group 4). The non-specific IFN-γ secretion might be attributed to the activation of NK cells by LPS contained in the OMVs, as it was reported that LPS could indirectly activate NK cells by activating DC or macrophages through LPS receptor TLR4 and triggering the production of NK stimulating cytokines and ligands (56). Substantial IgG level was also detected in OMVs as well as PBS control immunization group when examining the humoral immune response specific for rSpA antigen. The intrinsic ability of the Ig binding domains of rSpA to capture antibodies in sera may account for the high background IgG titer detected by the ELISA assay. Finally, OMV-Antigens generally outperformed OMV-Antigen cocktail in terms of activating antigen-specific cellular immunity while OMV-Antigen cocktail was better at evoking humoral immunity, implicating that distinct forms of the multicomponent click vaccine might favor different types of immune response *in vivo*.

Despite being more immunogenic than adjuvanted subunit vaccine, our click vaccines did not achieve high protection efficacy against Newman lethal challenge. Firstly, the selection of antigen is a fundamental determining factor for final protective efficacy that can be achieved. For three antigens we choose in this study, EsxA is a virulent factor interfering with host cell apoptotic pathways (57, 58). Sbi promotes the depletion of complement and inhibits opsonophagocytic clearance of *S. aureus* (59). SpA has high affinities and specificities to the complement binding (Fc) domain of immunoglobulins and also inhibits phagocytosis (60). Obviously, the combination of them when used as subunit vaccine was already less effective in the lethal challenge model (**Figure 5B**). Even though the OMV platform could significantly improve their immunogenicity, the improvement of protection efficacy might be trivial. Secondly, further optimization of the immunization routes as well as dosage may be necessary for the improvement of protection efficacy. Here, we choose the subcutaneous (s.c) route of immunization. OMVs can be preferentially uptaken by antigen presenting cells for processing due to their nano-size effect. Thus, Langerhans cells, the professional APCs situated under the skin can efficiently uptake OMV-based vaccines. S.c administration can also target the therapeutics to lymph nodes and the lymphatic system (61), where OMV-based vaccines can be further accumulated for immune stimulation. It is intriguing to test other immunization routes as they can not only affect the strength of the immune response, but also control the type of it. S.c, Intraperitoneal (i.p) or intramuscular (i.m) route may evoke different level of IgG and T cell based response that are important for *S.aureus* protection. It was reported that immunization of OMV vesicles through intranasal (i.n) route could additionally induce a significant level of IgA (62). Considering the recent finding of IgA in suppressing the multiplication of *S.aureus* (63), it is also interesting to check if i.n administration can improve protection, particularly in mucosal infection models.

## Data availability statement

The raw data supporting the conclusions of this article will be made available by the authors, without undue reservation.

## Ethics statement

The animal study was reviewed and approved by the Institutional Animal Care and Use Committee the Committee on the Use of Live Animals in Teaching and Research of the University of Hong Kong.

## Author contributions

JH, JS and NZ conceived the study. JS, XL and YH performed the experiments. NZ and JS analyzed the results, prepared the figures and wrote the manuscript. BZ helped in data analysis. JH revised the manuscript. All authors contributed to the article and approved the submitted version.
