# Supplementary material for: A bacterial outer membrane vesicle-based click vaccine elicits potent immune response against Staphylococcus aureus in mice
Source: Front Immunol. 2023 Jan 19;14:1088501. doi: 10.3389/fimmu.2023.1088501 (PMC9892643; doi:10.3389/fimmu.2023.1088501)
Supplement: Supplementary file 1 [file DataSheet_1.pdf]

# *Supplementary Material*

## 1 Supplementary Figures and Tables

### 1.1 Supplementary Figures

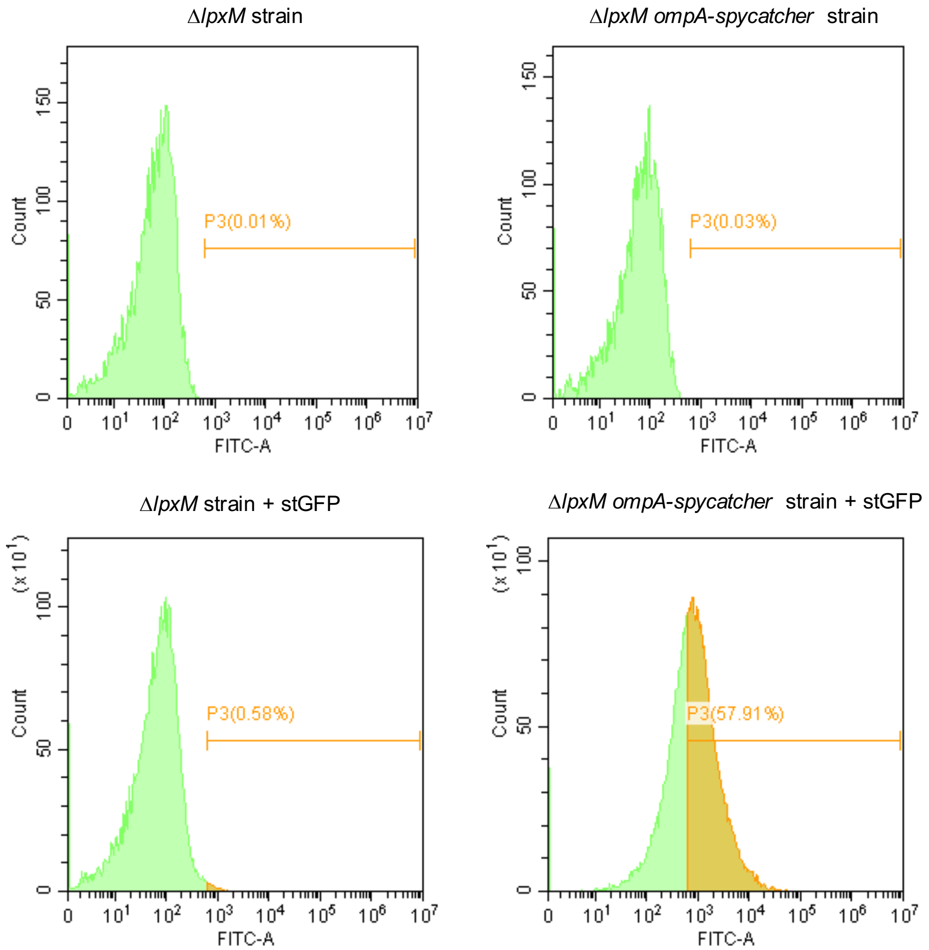


**Supplementary Figure 1. Flow cytometry analysis of the assembly between *ΔlpxM* or *ΔlpxM ompA-spycatcher* strains with stGFP.** Bacteria were collected and washed with PBS. Bacterial cell number for each group were adjusted to the same colony forming units (CFU) according to the $OD_{600}$. Bacteria were incubated with or without 5 μg stGFP at room temperature for 10 min. All samples were then washed three times with ice-cold PBS. The samples were subjected to flow cytometry analysis using FITC channel to detect the fluorescence.

### 1.2 Supplementary Tables

**Supplementary Table 1. Bacterial strains and plasmids used in this study.**

| Strain Number | Genotype/Phenotype | Source |
|---|---|---|
| MG1655 | Wild type *Escherichia coli* | Laboratory stock |

| | | |
|---|---|---|
| JS09 | MG1655 Δ*lpxM* | This study |
| JS72 | MG1655 Δ*lpxM ompA-spycatcher::kan* | This study |
| pJS59 | pET28a-spytag-gfp, Kan+ | This study |
| pJS73 | pET28a-spytag-sbi, Kan+ | This study |
| pJS74 | pET28a-spytag-esxA, Kan+ | This study |
| pJS79 | pET28a-spytag-spA, Kan+ | This study |

**Supplementary Table 2. Primers used in this study.**

| Primers | Sequences |
|---|---|
| dellpxM-1 | CACCAGATTGATTTTTGCCTTATCCGAAACTGGAAAAGCGCCGATCATATTCAATAACC |
| dellpxM-2 | TTATCATCAGGCGAAGGCCTCTCCTCGCGAGAGGCTTTTAAGCTTATCGATACCGTCGA |
| Colony-lpxM-1 | TAAACCAGCAGGCCGTAAAC |
| Colony-lpxM-2 | CATCCGGCCTACAGTTCAAT |
| ompASC-1 | GTGGACCAACAACATCGGTGACGCACACACCATCGGCACTGGCGGCGGCGGCAGCGGCGCCATGGTTGATACCTT |
| ompASC-2 | GGTCGACGGATCCCCGGAATTTAGTGGTGGTGGTGGTGGTGGTCAATATGAGCGTCACCTT |
| KN-1 | ATTCCGGGGATCCGTCGACC |
| KN-2 | GGTAGGAAACACCCAGGCTCAGCATGCCGTTGTCCGGACGTGTAGGCTGGAGCTGCTTCG |
| Colony-ompA-1 | GTTTCCGCGATTCTCTTCTG |
| Colony-ompA-2 | TAATGCGGAACACCAGCATA |
| ST-gfp-1 | TACAAGCCGACGAAGGGATCCGGCGGCGGCGGCAGCCGTAAAGGCGAAGAGCTGTT |
| ST-gfp-2 | GTGCGGCCGCAAGCTTGTCGACTTTGTACAGTTCATCCATAC |
| ST-esxA-1 | TACAAGCCGACGAAGGGATCCGGCGGCGGCGGCAGCGCAATGATTAAGATGAGTCC |
| ST-esxA-2 | GTGCGGCCGCAAGCTTGTCGACTTGCAAACCGAAATTATTAG |
| ST-sbi-1 | TACAAGCCGACGAAGGGATCCGGCGGCGGCGGCAGCAACCCAGACCGACGTGTTGCA |
| ST-sbi-2 | GTGCGGCCGCAAGCTTGTCGACTACAAAGCGGTTGTTTTTATC |
| ST-SPA-1 | TACAAGCCGACGAAGGGATCCGGCGGCGGCGGCAGCGCAAATGCTGCGCAACACGATGAAGCTAAAAA |
| ST-SPA-2 | GTGCGGCCGCAAGCTTGTCGACTTTCGGTGCTTGAGATTCGT |
